# Psychrophilic Bacterial Phosphate-Biofertilizers: A Novel Extremophile for Sustainable Crop Production under Cold Environment

**DOI:** 10.3390/microorganisms9122451

**Published:** 2021-11-28

**Authors:** Asfa Rizvi, Bilal Ahmed, Mohammad Saghir Khan, Shahid Umar, Jintae Lee

**Affiliations:** 1 Department of Botany, School of Chemical and Life Sciences, Jamia Hamdard, Hamdard Nagar, New Delhi 110062, India; asfarizvi09@gmail.com (A.R.); sumer@jamiahamdard.ac.in (S.U.); 2 School of Chemical Engineering, Yeungnam University, Gyeongsan 38541, Korea; 3 Department of Agricultural Microbiology, Faculty of Agricultural Sciences, Aligarh Muslim University, Aligarh 202002, India; khanms17@rediffmail.com

**Keywords:** abiotic stress, psychrophiles, phosphate solubilizers, crop nutrition, plant growth regulators, molecular engineering

## Abstract

Abiotic stresses, including low-temperature environments, adversely affect the structure, composition, and physiological activities of soil microbiomes. Also, low temperatures disturb physiological and metabolic processes, leading to major crop losses worldwide. Extreme cold temperature habitats are, however, an interesting source of psychrophilic and psychrotolerant phosphate solubilizing bacteria (PSB) that can ameliorate the low-temperature conditions while maintaining their physiological activities. The production of antifreeze proteins and expression of stress-induced genes at low temperatures favors the survival of such organisms during cold stress. The ability to facilitate plant growth by supplying a major plant nutrient, phosphorus, in P-deficient soil is one of the novel functional properties of cold-tolerant PSB. By contrast, plants growing under stress conditions require cold-tolerant rhizosphere bacteria to enhance their performance. To this end, the use of psychrophilic PSB formulations has been found effective in yield optimization under temperature-stressed conditions. Most of the research has been done on microbial P biofertilizers impacting plant growth under normal cultivation practices but little attention has been paid to the plant growth-promoting activities of cold-tolerant PSB on crops growing in low-temperature environments. This scientific gap formed the basis of the present manuscript and explains the rationale for the introduction of cold-tolerant PSB in competitive agronomic practices, including the mechanism of solubilization/mineralization, release of biosensor active biomolecules, molecular engineering of PSB for increasing both P solubilizing/mineralizing efficiency, and host range. The impact of extreme cold on the physiological activities of plants and how plants overcome such stresses is discussed briefly. It is time to enlarge the prospects of psychrophilic/psychrotolerant phosphate biofertilizers and take advantage of their precious, fundamental, and economical but enormous plant growth augmenting potential to ameliorate stress and facilitate crop production to satisfy the food demands of frighteningly growing human populations. The production and application of cold-tolerant P-biofertilizers will recuperate sustainable agriculture in cold adaptive agrosystems.

## 1. Introduction

Globally, abiotic stresses, including extremely low temperatures, cause major losses to the growth and productivity of food crops [1,2,3]. Indeed, low temperature is the most vital environmental variable which adversely affects composition, diversity, community structure, and microbial biomass, and decreases the soil nutrient pool [4,5,6,7]. Among soil microbes, the phosphate-solubilizing activities of phosphate-solubilizing bacteria (PSB) are negatively affected by many factors such as temperature, pH, salinity, and dissolved oxygen [8,9,10]. Of these, the low temperature restricts the cellular activities of microbes, where a 10 °C decrease in the temperature has been found to bring a 2–4-fold reduction in microbial enzyme activity [11]. Bacteria belonging to different genera, however, tolerate cold temperatures by synthesizing ice-binding proteins such as antifreeze protein (AFPs) which help them to survive and proliferate under freezing temperatures by regulating the formation and growth of ice crystals [12,13]. Literature in contrast also suggests that temperature, apart from impacting various metabolic activities of bacteria, also affects the P-solubilizing abilities of some psychrotolerant bacteria strains [14,15]. Due to this, attention in recent times has been paid to discovering PSB which can reduce/replace the use of chemical phosphatic fertilizers while protecting the plants from abiotic stresses. Psychrophiles among variously distributed microflora are the organisms that dwell in unpleasant conditions such as low-temperature environments, which, in general, are disastrous for most living organisms. Besides surviving extremely low temperatures, the “psychrophiles” also secrete active biomolecules capable of stimulating plant growth in low-temperature ranges [16,17,18]. Besides microbes, low temperatures negatively affect the morpho-anatomical, chemical, functional, and genetic composition of plants and, hence, decrease crop productivity [12,19]. The use of plant beneficial psychrophiles/psychrotolerant bacteria provides a sustainable option for optimizing agriculture production and offsetting the undesirable impact of cold temperatures [20,21]. The variations in plant growth-regulating abilities permit such organisms to thrive in different ecological habitats. In this regard, psychrophiles/psychrotolerant endowed with phosphate solubilizing ability, together with other plant growth-promoting potentials [22], have received greater attention and have gained impetus due largely to their ability to provide macro nutrients especially P to plants, ability to alleviate cold stress, and ability to modulate phytohormones growing under extreme cold soils [23,24]. Apart from providing P, psychrophilic PSB facilitate the growth of plants by providing phytohormones [12] and siderophores [2], or through phytopathogenic control [25] by secreting various cell wall degrading hydrolytic enzymes, for example, chitinases, proteases, and cellulases [2]. A study by Yadav et al. [17] reported the secretion of cold-active (at 4 °C) lytic enzymes such as amylase, β-glucosidase, pectinase, protease, cellulase, xylanase, β-galactosidase, laccase, chitinase, and lipase by psychrophilic, psychrotrophic, or psychrotolerant bacteria such as *Bacillus*, *Desemzia*, *Exiguobacterium*, *Jeotgalicoccus*, *Lysinibacillus*, *Paenibacillus*, *Planococcus*, *Pontibacillus*, *Sinobaca*, *Sporosarcina*, *Staphylococcus*, and *Virgibacillus*. The complex physiological process of psychrophilic PSB is, however, influenced by biotic or abiotic factors such as soil nutrient pool, soil pH, salt contents, temperature, and humidity [26]. These bacteria, when applied, have been reported to accelerate crop production under a low-temperature environment. For instance, the cold-tolerant PSB *Pseudomonas* isolates (RT5RP2 and RT6RP) colonizing the rhizoplane of wild grass, growing at 3100 and 3800 m above sea level, grew at a temperature that varied between 4 and 30 °C and when applied with Udaipur rock phosphate (URP) the P uptake of lentil plants under greenhouse conditions was significantly enhanced [27]. Considering these facts, attention in research is growing to discover cold-adapted PSB which could successfully colonize cold habitats and influence the processes of nutrient turnover at low temperatures. Thus, cold-adapted bacteria can be used as biofertilizers, biocontrol agents, and bioremediation for augmenting the growth and yield of crops growing at high altitudes [28]. The work presented is mainly a review of the recent advances in the novel field of cold-tolerant PSB which has direct relevance in crop optimization and yield stability under cold environments. This manuscript focuses on how cold temperatures affect the physiological activities of plants and briefly explains how plants alleviate cold stress. The ability of microorganisms to cope with the cold and to endure low temperatures, the mechanism of solubilization/mineralization of insoluble phosphorus, and how cold-active phosphate solubilizing bacteria promote plant growth under cold environments are discussed. Molecular engineering of phosphate solubilizing/mineralizing bacteria to broaden the host range is discussed. The work presented in this manuscript is likely to generate wide interest within the scientific community concerned for psychrophiles, psychrophilic PSB, and food production in cold regions.

## 2. Psychrophiles and Psychrotrophs: An Overview

### 2.1. Definition, Ecological Habitats, and Agronomic Importance

The cold regions cover approximately 71% of the Earth’s surface and 90% of the ocean volume is below 5 °C followed by snow, permafrost, sea ice, and glaciers [29]. Other cold environments are cold-water lakes, cold soils, cold deserts, and caves. Interestingly, these cold habitats are colonized by a variety of extremophiles including those possessing plant growth-promoting activities [16,22]. Such cold adaptive microorganisms have been categorized into two groups. (i) Psychrophiles: microbes which grow at or below 0, 15, and 20 °C (minimum, optimum, and maximum, respectively) and that can thrive well under identical environmental conditions [29,30]. Indeed, the psychrophily explains the capability of an organism to generate higher biomass at 15 °C or below instead of demonstrating better growth rates at temperatures over 20 °C [29]. (ii) Psychrotolerants or psychrotrophs: organisms capable of growing sub-optimally at temperatures below 20 °C or even at 0 °C that repel the negative effect forced by cold environments; such microbiota are recovered most frequently from cold environments. Since both groups of microorganisms can colonize cold habitats, the term “cold-active microorganisms” may be used at places in this manuscript to refer to both these groups. Sadly, efforts to understand the behavior and functional properties of such microbiomes under differing cold environments and the information available on their role in agronomic production in cold agroecosystems have been poorly described. Among agronomically potential organisms, cold-active phosphate solubilizing bacteria, for example, *Pseudomonas*, have been recovered from glacial ice habitats [18] and other cold ecosystems worldwide such as Andean mountain glaciers [31,32], polar environments [33,34], cold deserts [35,36], Himalayan soils [27,37], and alpine soils [38,39]. Some notable plant growth-promoting bacteria inhabiting the extreme cold environments are *Pseudomonas* [40,41], *Serratia* and *Staphylococcus* [42], *Exiguobacterium* [43], *Rahnella* [44], *Stenotrophomonas* and *Leucobacter* [45], and *Flavobacterium* [46]. Cold active microbes have prompted researchers to discover new cold-tolerant bacterial species possessing plant growth-promoting abilities [17,43,47], to produce cold-resistant enzymes [48,49] and mitigation of cold effects in plants in agroecosystem to optimize crop production [45]. Although reports indicating the functional response of plant growth-promoting rhizobacteria (PGPR) strains at low temperatures are available, studies on the P dissolving activity of soil microbiome at low temperatures (≤10 °C) are scarce. Despite this, Dolkar et al. [40] documented the potentiality of cold-tolerant P-solubilizing *P. simiae*, recovered from the Seabuckthorn rhizosphere, in plant growth promotion at low-temperature conditions. They observed a significant increase of 30% and 51% in shoot and root length in *P. simiae* inoculated tomato grown in pots maintained in the greenhouse, and 9.8% and 19.8% in open field conditions, respectively. The rhizobacterium *P. putida* GR12-2 recovered from the plant rhizosphere of the Canadian high arctic grew well and enhanced root growth of spring and winter canola at 5 °C at which very few bacteria could grow and function normally. Also, the bacterium had one major protein (32–34 kDa) and several minor proteins which benefit bacteria survival at −20 and −50 °C [50]. Similarly, psychrotolerant PSB *Pseudomonas* isolates (RT5RP2 and RT6RP) collected from the rhizoplane of wild grass growing at 3100 and 3800 m above sea level survived well between 4 to 30 °C and produced both IAA and siderophores at 4 °C. Under pot culture conditions, the psychrotolerant *Pseudomonas* strains in the presence of Udaipur rock phosphate (URP) significantly enhanced the vegetative growth (root and shoot), grain yield plant^−1^ by 20.9% and 13.8%, respectively, and P uptake by lentil plants relative to the sole application of RP [27].

### 2.2. How Do Cold-Active Bacteria Survive under Cold Stress?

To thrive well under cold extremes and to protect themselves from cellular injuries or to reduce the destructive effects of ice crystal formation, generally termed “Osmoprotection”, microorganisms have developed various structural and functional modifications [51,52]. These adaptations include (i) variation in membrane fluidity [53]; (ii) conformational flexibility; (iii) better enzyme activity associated with essential cellular processes such as transcription and translation [54]; (iv) induction of cold-shock proteins [55]; (v) production of antifreeze proteins (AFPs) [56,57]; (vi) excretion of high amounts of exopolymeric substances with cryoprotection activity [58,59,60,61]; (vii) synthesis and accumulation of various other compatible solutes such as polyamines, sugars, polyols, amino acids, etc. [62,63]. Together, the metabolic adaptation and the capability to grow widely determine the ecological success of psychrophilic bacteria under cold environments [64].

At cold temperatures, the membrane of cold-active bacteria becomes significantly more inflexible, which activates a membrane-bound sensor. The sensor so generated then produce the signals in response to the regulator, which ultimately upregulates the expression of genes associated with membrane fluidity and eventually upregulates different genes that assist bacteria to adapt to cold [65]. The presence of specific lipid constituents in the cytoplasmic membranes of cold-active bacteria helps to sustain fluidity and allows the transport of biomolecules through the membranes under low temperatures [66]. Also, psychrophiles secrete enzymes that can be extremely active at low and moderate temperatures compared to those produced by their mesophilic counterparts. The cold-active enzymes, which are heat labile, preserve the proper movement of the active site even at temperatures at which the molecular motions of mesophilic and thermophilic counterparts are critically limited [67,68]. In addition, psychrophiles/psychrotrophs bacteria enduringly synthesize one set of ice-binding proteins (AFP) at low temperatures whose concentration increases with rising low temperatures [69,70]. The AFPs reduce the freezing point of water without altering its melting point and eluding the formation of ice crystals [69,71]. Conclusively, the AFPs and accumulation of other compatible solutes, for example, glycine betaine as produced by *Bacillus subtilis*, prohibit ice-crystallization and as an effective cold stress protectant, respectively, and therefore allow bacteria to grow normally at growth-inhibiting temperatures [12,13,62]. Besides such stress alleviation mechanisms, the cold-active bacteria also synthesize and secretes exopolymeric substances, for example by *Colwellia psychrerythraea* 34H [60], which binds to bacterial cell surfaces and facilitates the production of the biofilms, entraps the nutrients, facilitates biochemical interaction, and guards the cells against hostile conditions [72]. The defense against reactive oxygen species (ROS) is yet another pivotal means by which cold-adapted bacteria thrive well at low temperatures since, if not protected, ROS can damage the bacterial cell structures under cold ecosystems significantly. The psychrophilic strategies to avoid the lethal impact of ROS include the generation of antioxidant enzymes such as catalase, peroxidase, superoxide dismutases, dioxygen-consuming lipid desaturases, or the absence of ROS-producing pathways [73,74,75]. Despite all of this, there is still a greater need to uncover the cold adaptation strategies adopted by cold-active microbes, which of course can be revealed through more and more metagenomic and proteomic technologies. These techniques can decipher the finer details and may provide precious information on the mechanisms of cold adaptation by the psychrophiles/psychrotrophs.

## 3. Low Temperature Effects on Plants: Physiological Changes and Stress Alleviation

With the consistently growing human populations, there is greater pressure on agrosystems to fulfill human food demands, which, however, are under severe threat due to some aggressive environmental conditions such as extreme low [76,77,78] and high [79,80,81] temperatures, drought [82,83], and salinity [84]. Temperatures below 15 °C (chilling stress) can have a detrimental impact on the growth and development of many food crops, including cereals like rice [19,85], maize [86,87], wheat [88,89], vegetables [90,91,92], legume crops [93,94], and many other plants [77,95]. At low temperatures, the cellular, physiological, and metabolic processes of plants are perturbed due to the induction of morphological and biochemical changes in plant tissues [78,96,97]. During the negative response, cold initially impairs the germination of seeds and causes poor stand establishment [98]. Following this, the critical physiological processes of plants, such as water metabolism [99], photosynthesis [100,101], nutrient metabolism [102,103], membrane lipids [104], proteins, and nucleic acids [105,106], are adversely affected, leading to the death of cells. Mechanistically, the low temperatures damage the cell membranes by blocking the water movement by altering stomata and disrupting the balance of cells metabolism [107,108,109]. Also, low temperatures reduce cellular respiration [110] and produce reactive oxygen species (ROS) in plants [111]. The ROS so generated at low temperatures can destruct the lipid profile of the membrane, proteins, and nucleic acid, which either alone or simultaneously leads to cell death [112,113]. When the membrane is damaged due to osmotic imbalance, the secretion of osmolytes, soluble proteins, and proline is enhanced, which eventually reduces the ability of cells to obviate the cold stress [114] and may cause the death of the cells [115]. As an example, extreme temperatures have been found to negatively upset the growth and many developmental processes of rice, such as germination, emergence and seedling establishment, and reproductive and grain-filling stages. The low (cold) temperature slowed down the vegetative growth; decreased the seedling vigor, number of seedlings, and tillering; and eventually enhanced the chances of plant mortality [116,117]. These results are supported by the findings of other researchers who also observed a similar deleterious impact of low temperatures on many food crops (Table 1).

In order to alleviate cold stress, plants have evolved mechanisms such as the formation of stress proteins [128], organic osmolytes, and phytohormones such as ABA, GA, brassinosteroids (BR), jasmonates (JA), auxin, cytokinin (CK), melatonin, and polyamines [74,129]. Also, they have antioxidant enzymes, for example, SOD, POD, CAT, and APX [130,131], and temperature-induced non-enzymatic active biomolecules to circumvent the ROS-induced damage, which is vital to maintain the redox balance of the cells [132,133,134]. Apart from these, certain compatible solutes generally referred to as “osmotic protectors” secreted under cold stress by plants is yet another imperative strategy adopted by plants to overcome abiotic stress [135,136]. For instance, soluble proteins, proline, and total free amino acids released by plants at low temperatures serve as osmotic protectors and guard plants against stress [101,137]. Of these cold-induced phytocompounds, the function of proline in cold management and its significance in shielding plants from cold stress has been well investigated [138,139]. Physiologically, proline can serve as a rapidly available source of N and C and assist plants during the phase of recovery from cold stress [140]. Proline may also act as a metabolite and a signal molecule and therefore affects plant development. Moreover, proline stimulates the secretion of some vital proteins and plays an important role in maintaining the expansion of cells under stress conditions [141,142]. The impact of temperature, i.e., both chilling (0–15 °C) and freezing (<0 °C), however, vary from genotypes to genotypes of plants.

## 4. Importance of P and Rationale for Using Cold-Active Bacterial Phosphate Biofertilizers in Low-Temperature Environments

Phosphorus is a vital nutrient that supports the growth and development of plants dramatically but its deficiency limits the crop severely [143,144]. Following uptake by the root systems and translocation to various plant organs, soluble phosphorus influences root morphogenesis, cellular growth, development of new tissues, macromolecular biosynthesis, respiration, signal transduction, energy transfer, and photosynthesis [145,146,147,148]. Although global soils have enough reserves of P (inorganic and organic P), very small amounts of the total soil P are accessible for plants [149] because of its rapid fixation/complex formation ability with Fe and Al in acid and alkaline soils, aggravating the nutrient problems of colder agrosystems [150,151,152]. To balance the P availability and to support the growth and development of plants, phosphatic fertilizers are repeatedly applied in agronomic practices [153,154,155]. However, due to the cost of its production and unpredicted environmental hazards like water eutrophication, sustainability of soil–plant systems, and human health problems, the use of chemical P fertilizers are discouraged [156]. Soil microbiomes having P solubilizing/mineralizing activity—often termed phosphate solubilizing microorganisms (PSM) in this context—have been suggested as a substitute to chemical phosphatic fertilizer [157,158] that transform the locked P into soluble forms which are taken up by plants [159,160]. To harness their potentials, phosphatic, nitrogenous, and potassic biofertilizers [161,162,163] have been produced and are commercially sold worldwide but their use in colder regions have been found to be grossly unproductive [164] due largely to the lethal impact of low temperatures on the functional activity of mesophilic homologs. As an example, the microbial enzyme activity decreases two to four times more when the temperature of any environment drops by 10 °C [11]. In addition, the poor availability of nutrients, low moisture and organic matter, minimal land, and harmful impact of low temperatures on crops are the major constraints that further worsen crop production in colder/high altitude agrosystem conditions that experience chilling temperatures. So, realizing the colder region problems and pressure on the agroecosystems, there is an imperative requirement to produce effective phosphate biofertilizers consisting of plant beneficial psychrotolerant or psychrophiles. The usefulness of such bacterial strains in cold-adapted agroecosystems (e.g., hill and mountain) seems huge due to the exceptional crop-raising conditions and the environmental situations of the high altitude agricultural systems [165,166]. The cold-adapted P biofertilizers could resist/tolerate the extremities of cold and maintain their functional qualities even under cold environments [167]. Considering the vast and varied plant growth-promoting potentials, the application of indigenous phosphate biofertilizers in cold soils can be a central approach to preserve soil fertility, protect microbial diversity, and concomitantly optimize crop production more sustainably.

## 5. Mechanisms of P-Solubilization in Cold-Adapted Bacteria: An Overview

Generally, the phosphate-solubilizing bacteria including psychrophiles [2,20], mesophiles [168,169], or thermophiles [156,170] convert the inaccessible complex P such as Ca_3_(PO_4_)_2_, Fe_3_PO_4_, and Al_3_PO_4_ to plant-available forms by acidification, chelation, exchange reactions, and polymeric substance formation [171,172]. On the other hand, the organically bound P (e.g., phytin, phospholipids, nucleic acid, etc.) is transformed into bioavailable forms by microbial enzymes, and, later on, the soluble P is absorbed by plant roots [173,174,175]. Like the mesophilic counterparts, the organic acid (OA) theory of P solubilization by cold-active bacteria is the most widely accepted mechanism of P availability in soil (Table 2). For example, different cold-active bacteria like *Pseudomonas* sp., *P. palleroniana, P. proteolytica,* and *P. azotoformans* while growing at 15 °C and 25 °C released mainly oxalic and malic acids, whereas the culture supernatants of these bacteria had a poor quantity of lactic, citric, and succinic acids [20]. In general, the P solubilization by psychrotolerant *Pseudomonas* sp. was maximal at 15 °C except for *P. azotoformans*, which showed maximum P solubilization at 25 °C. Likewise, gluconic acid, acetic acid, oxalic acid, quinic acid, and succinic acid are secreted by other PSB while growing under in vitro conditions [176,177]. The membrane-bound enzyme, for example, glucose dehydrogenase (gcd), mediates the synthesis of gluconic acid [178,179], an important organic acid causing solubilization of insoluble P by such PSB. The OA so released diffuses through the membrane and strongly acidifies the cell surroundings, leading eventually to the discharge of P ions from insoluble inorganic P sources by H^+^ substitution for Ca^2+^ [180,181]. The acidic (lowering of pH) environment of the medium suggests the release of OA that occurs on the outer face of the cytoplasmic membrane via the direct oxidation pathway. Many reports have established a positive correlation between pH drop and soluble P concentration in the liquid culture medium. Since the solubilization of inorganic P largely depends on the membrane-bound enzymes, the low temperatures may intensely affect its efficacy under a mesophilic environment. So, such conventional PSB requires urgent bioprospecting especially for solubilizing enzymes so that they remain active and perform efficiently at low temperatures, like those performed by cold-active PSB [167]. The OA secreted either by cold-active PSB or conventional PSB into the culture supernatant can be detected by paper or TLC [182] or by HPLC [20,183]. Besides OA, the release of H^+^, production of chelating substances, and inorganic acids (sulfuric, nitric, and carbonic acids) have been suggested as some other alternative mechanisms of P solubilization by PSB, as observed in the case of P fixation in acidic soil [184]. To elaborate further, Illmer and Schinner [185] reported that 0 to 5000 µM of gluconic acid at pH 4 to 7 did not affect Ca-P solubility at pH > 6. Moreover, the total amount of P solubilized under in vitro environments did not correlate with the amounts of OA excreted into the supernatant suggesting the involvement of biomolecules other than OA in P solubilization by PSB. The inorganic acids, for instance, hydrochloric acid, can also solubilize insoluble P but in terms of solubilization, they have been poorly effective relative to those solubilized by OA at the same pH [186].

Enzymes like phosphatases, phytases [20,195], and phospholipases [196] produced by many psychrotolerant/conventional PSB induce the release of P from phosphorus-containing organic molecules (e.g., phospholipids, phytin, nucleic acid, etc.) through mineralization process in the rhizosphere soil. As an example, the cold-tolerant *Pseudomonas* sp. produced maximal amounts of phytase at 15 °C, whereas *P. azotoformans* excreted the highest quantity of phytase at 25 °C. The phytase so released by both the cold-tolerant PSB transformed the phytate (a natural soil organic P) into plant-available orthophosphate through the mineralization process [20]. Similarly, the genetically engineered root-associated mineral P solubilizing (*mps*) bacteria, *P. simiae* WCS417r, *Ralstonia* sp. strain UNC404CL21Col, and *P. putida* KT2440 caused the release of plant-available P from phytate [197]. Apart from this, the persistence of mineralizing activities of acid and alkaline phosphatase further confirms the ability of bacterial cultures to release P from organically bound P as also reported in *Serratia* sp. [198] and *Pantoea* sp. [173] by other workers.

## 6. Mechanisms Used by Cold-Adapted PSB to Facilitate Plant Growth

**Figure 1:**
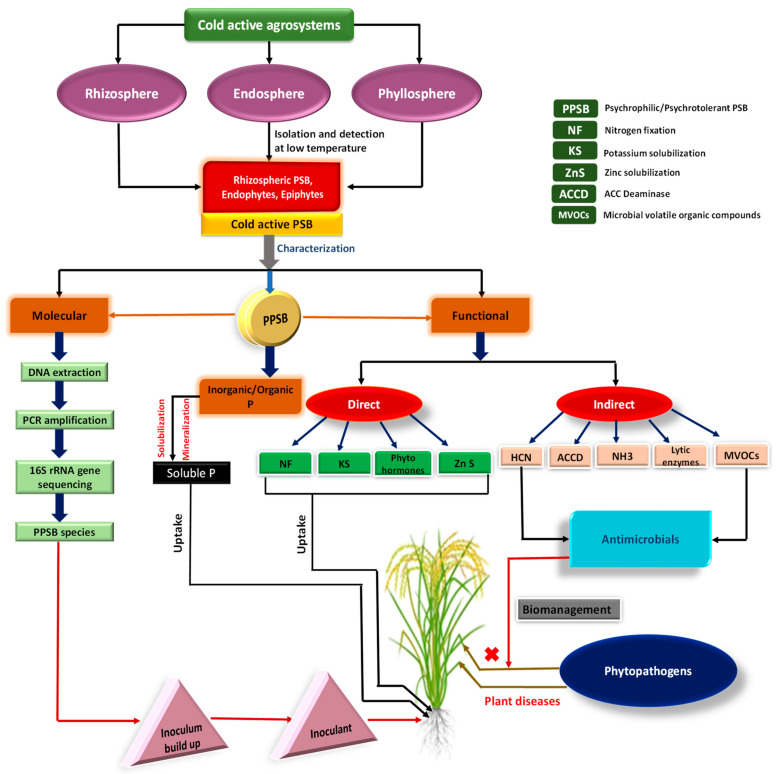
Cold active phosphate biofertilizers: isolation, characterization, P solubilization, and plant growth promotion in the low-temperature environment.

The concept of applying cold-adapted PSB among many cold-active PGPR makes it clear that the cold temperatures and poor growing seasons influence both versatile microbiomes and plants growing under cold/chilling temperatures. So, the discovery of novel cold-active PSB, which maintains their plant growth-promoting traits even under low-temperature environments and counterbalances the damaging effect of cold temperature, is indeed essentially needed to optimize food production in colder areas of the world. The mechanisms used by conventional PSM including bacteria, fungi, and actinomycetes to optimize crop production in different agroecosystems have been previously reviewed [172,199,200]. However, the mechanisms adopted by psychrophiles/psychrotrophs PSB to ameliorate crop production in a low-temperature environment have perhaps not been reviewed due largely to scanty literature on this aspect. Despite this, the literature available here and there on cold-active PSB including rhizosphere bacteria, epiphytes, and endophytes able to exhibit direct or indirect ameliorative effects on plants (Figure 1) are reviewed and discussed.

### 6.1. Direct Mechanisms

Epiphytic, endophytic, and rhizospheric cold-active PSB bacteria promote plant growth directly by the expedition of resource and nutrient acquisition, primarily the solubilization and mineralization of inorganic and organic P, respectively [20], a mechanism also employed by other mesophilic homologs PSM [175,201], fixation of atmospheric N, solubilization of potassium and zinc, secretion of siderophores and phytohormones such as cytokinin, auxin, and gibberellins [12,37,202,203]. The phyto-beneficial active molecules identified in cold-active PSB supporting plant growth, however, differs from organism to organism and from temperature to temperature (Table 3). Cold tolerant PSB, for example *P. fluorescens* [204], *P. lurida* M2RH3 [205] and *A. chroococcum* [206], also produce siderophores that solubilize and form a complex with iron in the rhizosphere and, therefore, offer a survival advantage to both plants and bacteria by removing phytopathogens and other microbial competitors through iron limitation [2,43]. The secondary metabolites released by psychrophilic PSB strains could therefore serve as attractive and useful biotechnological tools in reducing the damage to plants caused by the attack of a variety of pathogens [18].

Nitrogen-fixing microorganisms can transform the atmospheric N_2_ into ammonia and provide it to plants. Like traditional N_2_ fixers, the uptake of nitrogen fixed by psychrotolerant bacterial species of *Arthrobacter*, *Bacillus*, *Bordetella*, *Providencia*, *Pseudomonas*, *Acinetobacter,* and *Stenotrophomonas* facilitates plant growth and increases the nutritional value of crop plants [213]. In a similar study, Zhang et al. [217] reported that the rhizobia recovered from the cooler climates of North America markedly affected symbiotic features, such as N_2_ fixation and nodulation of soybean, relative to the rhizobia isolated from the warmer southern climes. The production of stimulatory phytohormones by cold-active PSB including IAA, cytokinin, gibberellin, abscisic acid, etc. [22,35,36] is yet another direct mechanism of plant growth stimulation. Of these hormones, the IAA directly affects morphogenesis including root primary growth, side root formation, and root hairs. Many cold-tolerant PSB are capable of secreting IAA at low temperatures, including *Pantoea dispersa* and *Serratia marcescens* SRM [218] and *Pseudomonas jesenii* strain MP1 [219], have been isolated from cold environments. Similarly, the psychrotrophic PSB recovered from the cold desert of the Himalayan region produced gibberellic acid (GA) [35,36]. However, some cold-active PSB, for example, *Stenotrophomonas*, can produce more than one hormone (e.g., IAA and GA) which either alone or simultaneously facilitates the development of plants [213]. The combined effect of such valuable biomolecules on plants is usually measured by evaluating the cumulative plant growth, nutrient uptake, and total biomass production [220].

In addition to producing phytohormones, many cold-tolerant PSB, for instance, *Pseudomonas, Serratia,* and *Flavobacterium*, possess the enzyme ACC deaminase [12] which can lower plant ethylene (C_2_H_4_ or H_2_C=CH_2_) levels [221]. Ethylene, a phytohormone synthesized under biotic/abiotic stress [222] induces senescence, chlorosis, and abscission in plants, thereby aggravating the lethal impact of pathogens [223,224]. The enzyme ACC deaminase cleaves ACC, a precursor of ethylene formation, into α-ketobutyrate and NH_3_ [225] and, therefore, decreases the levels of ethylene. Once the plants have decreased levels of ethylene, they grow normally under stress. As an example, ACC deaminase-producing psychrotolerant bacterium *P. putida* UW4 facilitated the bio-performance of canola plants growing at low temperatures [226]. Several studies have validated the efficacy of the ACC deaminase enzyme produced by cold-active PSB to protect plants from attack by the phytopathogens [35,43].

### 6.2. Indirect Mechanisms

Cold adapted PSB can also stimulate the growth of plants indirectly by restricting the functioning and further multiplication of crop-damaging phytopathogens [203,207,227]. Due to this, the indirect mechanisms become of great practical significance because they avoids the use of synthetic pesticides in agronomic practices. Currently, although the technology for the large-scale production of biopesticides using mesophilic microbes is available, these biocides have not been active at low temperatures due to the destructive effect of cold temperatures on biocontrol agents. Hence, there is great interest in identifying the cold-active PSB possessing biocide activity for application in low-temperature environments [2,228]. Sadly, the reports on such cold-active microbiomes with biocontrol potentials are scanty. In this section, we review the current information available on the antagonistic potentials of cold-active bacteria.

Cold-adapted PSB produce hydrolytic enzymes such as chitinases and proteinase, which degrade the fungal cell walls and therefore protect the crop plants from attack by pathogens, reducing the necessity of using environmentally hazardous chemicals to optimize crop production [17,229,230]. For example, several psychrophilic *Pseudomonas* spp. strains through lytic enzymes protease have been shown to inhibit the phytopathogens *P. ultimum*, *F. oxysporum*, and *P. infestans*. Other hydrolytic enzymes such as chitinases and cellulases degrade cellulose and chitin, respectively. As a result, the cold-active PSB able to produce such enzymes improves colonization and protects plants from phytopathogens like *Phytophthora* and *Phytium* species whose cell walls contain cellulose and other fungi with a chitin cell wall [231]. Some other well-recognized phytopathogenic fungi include the genera like *Rhizoctonia, Alternaria*, and *Verticillium*, which cause serious damage to crops. So, the application of cold-adapted plant growth-promoting bacteria to prevent the attack of phytopathogens and hence to limit the progression of the diseases is imperative for the sustainability of agro-ecosystems [25]. In this context, four different strains of psychrophilic *Pseudomonas* impeded the growth of fungal phytopathogens, *Fusarium* sp., *R. solani*, *A. solani,* and *P. capsica*, by secreting chitinases and proteases [2]. Likewise, a chitinase-producing *Pseudomonas* sp. recovered from marine sediments suppressed the growth of two phytopathogenic fungi, *V. dahlia* and *F. oxysporum* f. sp. *cucumerinum* [232]. Production of cyanogenic compounds by cold-active PSB inhabiting various low-temperature environments [18,35,213] is yet another special trait that aids in the management of phytopathogens, although it is harmful to certain plants because it interferes with the cytochrome P450 system [233]. To counteract the cyanogen toxicity, plants possess a cyanide resistant respiratory pathway [234] but microbes do play a critical role in avoiding cyanogenic toxicity. For instance, seed inoculation with HCN positive phosphate solubilizing psychrotolerant *P. fragi* CS11RH1 had no damaging influence on germination or growth of wheat plants [14]. The ability of the wheat plant to counterattack cyanide action was due to cyanogenesis by *P. fragi*, which avoided cyanogenic toxicity, as also reported for the take-all disease of wheat caused by the fungal pathogen *Gaeumannomyces graminis* var. *tritici* [235]. Another indirect mechanism of growth stimulation by psychrophilic and psychrotolerant bacteria involves the production of gluconic acid which can control the populations of plant pathogens. Psychrophilic and psychrotolerant bacteria mainly belonging to class actinobacteria and proteobacteria collected from Andean glaciers produced high quantities of gluconic acid [31,32]. The majority of these cold-tolerant bacterial strains limited the growth of phytopathogenic fungi, *F. oxysporum* and oomycetes, *P. infestans*, and *P. ultimum*. Conclusively, the multifarious growth stimulatory activity of cold-tolerant PSB represents one of the most important functional groups of bacteria, which could be developed as suitable bacterial formulations able to endure at extremely low temperatures while retaining their plant growth modulating traits for crops growing in low-temperature environments.

## 7. Performance of Cold-Active P-Biofertilizers under the Low-Temperature Environment

Plant–microbe interactions are influenced by changes in temperature [22,236] wherein the low temperature among different abiotic stresses poses a major limitation on the growth and reproductive stage of development and grain yield of plants [237]. In this regard, cold-tolerant or low-temperature adapting plant growth-promoting P bacteria have an advantage over their mesophilic counterparts to increase growth and productivity, particularly in the areas perturbed by low temperatures. Also, the cold adaptive bacterial formulations have a greater possibility of substituting agrochemicals used to optimize crop production in nutrient/cold stressed soils. Understanding the crosstalk between cold-active bacteria and crops growing under cold regimes will, therefore, be needed to develop microbial strategies to protect plants from cold stress vis-à-vis augmenting their growth under low-temperature regions.

Psychrophilic and psychrotolerant bacteria recovered from cold environments [238,239] have demonstrated plant growth-enhancing activity both in greenhouse and field conditions that prompted the scientists to apply them in cold agricultural ecosystems also [27,240]. The Antarctic *Pseudomonas*, a psychrotolerant strain (Da-bac Ti8) possessing plant-growth modifying ability has previously been used as a psychrotolerant biofertilizer formulation and is currently patented as well [215,241]. The bacterial preparation (inoculant) of psychrophilic or psychrotolerant *Pseudomonas* spp. have demonstrated some promising results in many food crops like mungbean [242], wheat [194,205,216,243], maize [244,245], rice [246], and lentils [37,41]. In addition to the non P solubilizing cold-active plant beneficial bacteria, the low-temperature tolerant PSB applied in P deficient soils of colder regions have been found agronomically useful against many crops (Table 4). The use of cold-tolerant P-solubilizing bacteria in stressed agrosystems is recommended due to reasons such as (i) inexpensive production; (ii) the ability to tolerate other stressor molecules besides cold, without losing functional traits; and (iii) being environment friendly. Some notable phosphate solubilizing bacterial genera possessing stress tolerating ability include *Cronobacter* [247], *Azotobacter* [248], *Aerococcus*, *Pseudomonas* and *Pantoea* sp. [173], *Achromobacter* [249], *Bacillus* sp. [250,251]. Psychrotrophic PSB, *P.* *jesenii* MP1, and *Acinetobacter* sp. ST02 enhanced the germination of seeds by 92% (MP1) and 85% (STO2) and significantly increased the agronomical and biochemical parameters of chickpea grown under field conditions [252]. Of the two strains, strain MP1 applied with 40 kg P_2_O_5_ ha^−1^ had maximum impact on grain yield and harvesting index, indicating that the use of PSB with a recommended dose of P fertilizers is greatly beneficial for high altitude agriculture. Similarly, the cold-tolerant *P. corrugata* enhanced the yield of maize grown in greenhouse and fields in rain-fed conditions [244]. Rondón et al. [16] in yet another investigation observed that the *Pseudomonas* strains recovered from the Andean glacier enhanced the biological performance of wheat plantlets at low temperatures. Conclusively, the use of cold-tolerant PSB, individually or as a mixture, has been found effective in optimizing crop production under low-temperature regimes. Therefore, the cold-active PSB–plant interactions have great agronomic potential and promise to be an efficient tool for bioprospecting food production, even under cold stressed environments.

## 8. Molecular Engineering of Phosphate-Biofertilizers

Phosphate solubilizing bacteria surviving at mesophilic, thermophilic, or psychrophilic temperatures release plant-available P from both inorganic and organic P stores. Therefore, they may serve as sustainable and inexpensive alternatives to high-cost and environmentally hazardous chemical P fertilizers. However, many plant growth-promoting bacteria (PGPB) colonizing the root surface and thriving around the root systems do not release plant accessible P from soil reserves because they lack the capacity to solubilize/mineralize the soil P. Therefore, the effort is directed to develop a pool of P-solubilizing soil bacteria that grow in the root region and could release plant accessible P. Recombinant technology in this regard offers a viable option to engineer non-P-dissolving soil bacteria to improve their P solubilizing/mineralizing ability that can be applied against wide-ranging crops [255,256]. Also, the engineered bacterial strains could be customized according to the crops and environmental conditions. Considering this, different molecular approaches have been used to genetically modify non-P-solubilizing plant beneficial bacteria to release plant-available orthophosphate from different organic P. Molecular engineering of PSB by insertion and over-expression of genes associated with soil P dissolution into non-solubilizer soil bacteria is indeed a promising approach for enhancing the capability of non-P-solubilizing bacteria and host range to be used as PSB inoculant [257,258]. Furthermore, cloning and transfer of P-solubilizing/mineralizing genes into non-PSB may avoid the need for mixing two phylogenetically and physiologically contrast bacterial populations, for example, nitrogen-fixers and P-solubilizers, as inoculants together, if the technology is successful. Molecular engineering focuses on transferring especially mineral phosphate solubilization (*mps*) genes or the enzyme encoding genes associated with the dissolution of organic P from solubilizing/mineralizing to non-P-solubilizing/mineralizing PSB. Some of these approaches are briefly discussed.

### 8.1. Development of mps Positive Bacterial Strains

Goldstein [259] suggested for the first time the existence of *mps* genes in a Gram-negative epiphyte *Erwinia herbicola,* currently known as *Pantoea agglomerans*. Applying the shotgun-cloning technique, Goldstein and Liu [260] cloned gene(s) associated with *mps* and demonstrated that the pyrroloquinoline quinone-linked glucose dehydrogenase (PQQGDH) mediated dissimilatory bypass system catalyzes the oxidative transformation of glucose to gluconic acid (GA) that occurred in the bacterial periplasmic space. The resulting GA caused the solubilization of mineral P in *E. herbicola*. Further insertion and subsequent expression of this *mps* gene into *E. coli* HB101 led to the synthesis of GA and imparted P solubilizing potentials to the non-PSB to solubilize hydroxyapatite (MPS^+^ phenotype). The *E. coli* strains lacking *mps* (MPS^−^) can produce GDH (encoded by *gcd)* but they cannot produce the PQQ—a cofactor encoded by the PQQ operon [261] for the GDH and, hence, may not produce GA [262]. In a similar experiment, *E. coli* produced higher amounts of GA and dissolved hydroxyapatite when the *mps* gene of *Ranella aquatilis* was integrated into the non P solubilizing recipient bacterium [263]. However, both bacterial species differed in the regulation of the *mps* genes. Similar efforts to increase the *mps* ability in conventional mesophilic plant growth regulators, for example, *B. cepacia* and *P. aeruginosa* strains through PQQ synthase genes obtained from *E. herbicola*, was done using a broad-host-range vector pKT230 [264]. Using the tri-parental conjugation process, the recombinant plasmid was allowed to express in *E. coil,* and thereafter it was inserted into two recipients cells of *B. cepacia* and *P. aeruginosa*. Many of the resulting clones possessing recombinant plasmids displayed a larger P solubilization zone on solid PVK medium containing insoluble TCP. The production of clear halo around the bacterial conjugants suggested a successful expression of *mps* activity of the *E. herbicola mps* gene in the non-P solubilizer PGPR strains. Expression of the *mps* genes in a non-P solubilizer, however, depends on the genetic composition of the recipient bacterial strains, the plasmid copy number, and metabolic reactions.

### 8.2. Development of Enzyme Engineered Bacterial Strains

#### 8.2.1. Development of Bacterial Strains with Phosphatase Activity

Besides inorganic phosphorus, the organically bound P [265] is mineralized to release free orthophosphate by enzymes acid phosphatase (encoded by *olpA*), alkaline phosphatase (*phoD*) [198], phytases (*appA*), phosphonatase (*phnX*), and C-P lyase (*phnJ*) [101,266,267,268]. Of these, phytases and phosphatases are the most common P mineralizing enzymes which have been transferred to non-P mineralizing bacteria. For example, the PhoC acid phosphatase gene of *Morganella morganii* (phoC gene) cloned using a vector was transferred through chromosomal integration into phoC negative PGPR strains of *Azospirillum* spp. and *B. cepacia* [269]. Similarly, a gene expressing phosphatase activity was isolated from *B. cepacia*. The *napA* phosphatase gene of *M. morganii* was integrated into *B. cepacia* IS-16 using a broad-host-range vector (pRK293). The recombinant strain demonstrated a considerable enhancement in phosphatase activity. In a similar study, the chromosomal insertion of a heterologous gene encoding an acid phosphatase enzyme in a putative PGPB is reported [270]. Briefly, The phoC gene of *M. morganii* that encodes for the acid phosphatase was cloned in the pJMT6 mini-Tn5 derivative transposon vector and the phoC gene was further integrated into the *P. putida* N-14 chromosome. The resulting *P*. *putida* N-14::Tn5-phoC expressed high levels of the enzyme providing the superfluous ability to the recombinant bacterium to mineralize P from organic compounds. However, even after chromosomal integration, the *P. putida* N-14 strain retained their original PGP potentials. The phosphatase activity of recombinant *P. putida* N-14 was also detected by SDS-polyacrylamide gel electrophoresis (PAGE). Analysis by SDS-PAGE revealed a band of approximately 25 kDa produced by *P. putida* N-14::Tn5- phoC, which was closely related to those reported for *M*. *morganii* acid phosphatase PhoC [271]. However, no band and, therefore, no phosphatase activity was detected in the native *P. putida* N-14 strain.

#### 8.2.2. Development of Bacterial Strains with Phytase Activity

Phytate (myo-inositol 1,2,3,4,5,6-hexakisphosphate) among organic P derived from plant, microbes and metazoan biomass predominates in soil [272]. Phosphate mineralizing bacteria such as *Advenella* spp. and *Cellulosimicrobium* sp. PB-09 produce phytase and affect the mineralization of organic P [273] while some bacteria may colonize the root surface or inhabit the rhizosphere regions but they can’t mineralize soil organic P and, therefore, fail to supply soluble P to growing plants. Despite this, such bacteria can be molecularly engineered to release P because they are more acquiescent to engineering and can be applied against a range of crops. With these backgrounds, Shulse et al. [197] using a combinatorial synthetic biology-based approach generated numerous plant-colonizing bacteria that could hydrolyze phytate. Overall, they produced 82 biochemically different phytase enzymes (encoded by phytase gene) and transferred them directly into the genomes of *P. simiae* WCS417r, *Ralstonia* sp. strain UNC404CL21Col, and *P. putida* KT2440 using conjugation techniques and observed that the engineered bacterial strains hydrolyzed the phytate very efficiently in liquid culture medium. Also, they released Pi from TCP probably due to the secretion of OA (s). Furthermore, many of these phytases secreting bacterial strains significantly promoted the growth of *A. thaliana* grown under Pi limited soilless conditions (agar plate assays). In another set of experiments, *Arabidopsis* plants bacterized with a total of 14 different host/gene combinations were grown using phytate as the only P source. The growth of *Arabidopsis* in the presence of phytate but without bacterial inoculum was consistently poor and plants showed deposition of anthocyanin. The accumulation of anthocyanin resulted in a dark-colored leaf in *Arabidopsis* plants, which suggested that the *Arabidopsis* did not take up sufficient Pi from phytate. In contrast, *Arabidopsis* plants treated with engineered bacterial strains accumulated greater dry matter and had bigger-sized rosettes relative to the uninoculated plants. The increment in the measured biological properties of *A. thaliana* was due to the availability of P supplied by the engineered bacteria which caused the enzymatic dissolution of phytate. Considering all of this, molecular engineering provides a promising opportunity to produce bacterial strains with enhanced P solubilization/mineralization ability and broader host range which could serve as efficient microbial inoculants for furthering agricultural production in low P soils under different environmental conditions including cold temperatures. Metagenomic and genomic approaches can help understand the phenotypic features considered important for their growth-promoting abilities at molecular levels [274]. Genomic and proteomic strategies will further help to establish a meaningful correlation between important secondary metabolites such as organic acids, enzymes, and other growth enhancers with genes and proteins that would reveal the overall plant growth modulating and low-temperature meliorative behavior of cold-adapted phosphate biofertilizers.

## 9. Conclusions and Future Prospects

Cold active phosphate solubilizing bacteria involving different genera like *Pseudomonas, Acinetobacter*, *Bacillus*, *Stenotrophomonas,* etc. has huge potential for modifying nutrient-deficient soils into nutrient sufficient soils under a low-temperature environment. The production of important food crops such as cereals, vegetables, and legumes can be optimized in colder areas using such cold-adapted PSB strains, thereby reducing the dependence on chemical fertilizers applied in the intensive agrosystems. Future research is required to decipher the molecular basis of growth-enhancing mechanisms and to understand how cold-active PSB strains retain their functional traits while thriving at low temperatures. The development of environmentally friendly cold-active phosphate biofertilizers opens up new horizons for enhancing crop production in colder regions.

## Figures and Tables

**Table 1:** Effect of low temperatures on biological and biochemical features of food crops.

Crops	Scientific Name	Growth Conditions	Applied Low Temperatures	Crop Responses	References
Soybean	*Glycine max*	Growth chamber	25 °C to 10 °C	At 10 °C germination was completely inhibited; very slow at 15 °C but germinated well at 25 °C; highest cell membrane permeability at 10 °C and 15 °C; at 10 °C, the dehydrogenase activity was highest but -α-amylase was poor at 10 °C; photochemical efficiency was higher in Malaga and Petrina plants germinating at 10 °C and 15 °C than at 25 °C; at a lower temperature, dry weight and number of pods reduced but the number of seeds was higher at 10 °C compared to 15 °C and 25°C; seed weight did not differ among temperatures	[98]
Peas	*Pisum sativum*	Greenhouse	4 °C to −20 °C	Proline content and activities of antioxidant enzymes such as APX, SOD, and CAT gradually increased at cold acclimation	[118]
Chickpea	*Cicer arietinum*	Field	≤10 °C	Vegetative growth was reduced and all the phenological stages were delayed; caused vegetative aberrations like chlorosis, necrosis of leaf tips and curling of the whole leaf; damage to reproductive stage involved abscission of juvenile buds and flowers and abortion of pods, pollen development was suppressed and seed formation was inhibited	[119]
		Pots	<20 °C/<10 °C (day/night)	Decreased chlorophyll content, relative leaf water content, dry weight, and yield features such as pods, seed number, and seed yield; increased electrolyte leakage, reduced total sugars, and starch, poor β-amylase, invertase and sucrose synthase; greater oxidative stress, poor levels of enzymatic antioxidants and reduction in proline and ascorbic acid	[120]
		Greenhouse	11.7/2.3 °C (day/night)	The chilling conditions increased electrolyte leakage, inhibited chlorophyll formation, decreased sucrose content, the water content in leaves, declined total plant weight, reduced the rate and duration of the seed filling, seed size, seed weight, pods per plant and harvest index, reduced the accumulation of starch, proteins, fats, crude fiber, protein fractions like albumins, globulins, prolamins, and glutelins; also, chilling declined the level of sucrose and enzymes such as starch synthase, sucrose synthase, and invertase significantly in the seeds; minerals such as Ca, P and Fe and amino acids were lowered significantly in the stressed seeds	[121]
Wheat	*Triticum aestivum*	Field air temperature control system (FATC)	5.3 °C to −7.0 °C	Low-temperature stress prolonged the growth period significantly decreased net photosynthetic rate, plant height, and biomass production, and reduced grain yield	[122]
Maize	*Zea mays*	Greenhouse	8 °C to 4 °C	Reduced germination, increased number of dead seeds, reduced plumule dry weight and radicle, declined the rate of metabolic activity	[123]
Rice	*Oryza sativa*	Greenhouse	22 °C to 14 °C	Significantly reduced shoot and root growth, physiological attributes, leaf chlorophyll fluorescence associated parameters, and dry matter production	[19]
		Field conditions	<15 °C	Increased the chlorophyll-a, chlorophyll-b, chlorophyll-a/b ratio, and total chlorophyll, increased proline concentration but decreased carotenoid content	[124]
Tomato	*Lycopersicon esculentum*	Greenhouse	14.6 °C	Suppressed fruit yield, restricted fruit mass, increased soluble carbohydrates, total amino acids, and guaiacol peroxidase activity in roots, leaves, and fruit, and superoxide dismutase in fruit but significantly lower malondialdehyde content	[125]
Potato	*Solanum tuberosum*	Growth chamber	4 °C/2 °C (day/night)	Soluble protein, MDA, and proline enhanced with low-temperature exposure duration but the chlorophyll content decreased; protein spots (N = 52) identified in proteomic studies were involved in defense response, energy metabolism, photosynthesis, protein degradation, ribosome formation, signal transduction, cell movement, N metabolism, and other physiological processes	[126]
Cabbage	*Brassica oleracea*	Pot trays under controlled conditions	12 ± 1 °C	Low temperatures affected photosynthesis and fresh weight; stomatal conductance and leaf water content were significantly reduced; plants had smaller but thicker leaves; chilling conditions did not show any reduction in the dry matter	[127]

**Table 2:** Organic acids secreted by cold-active phosphate solubilizing bacteria.

Cold-Active PSB	Ecological Habitat	Organic Acids	References
*Pseudomonas* sp., *Pseudomonas palleroniana, Pseudomonas proteolytica, Pseudomonas azotoformans*	Soils from high altitudes in Indian Himalayas	Oxalic, lactic, malic, citric, and succinic acids	[20]
*Serratia plymuthica*	Soils	Gluconic acid	[187]
*Bacillus*, *Burkholderia, Paenibacillus* sp.	Paddy field	Gluconic oxalic, citric, tartaric, succinic, formic and acetic acid	[188]
*Pantoea*, *Pseudomonas*, *Serratia*, and *Enterobacter*	Wheat rhizosphere	Oxalic, citric, gluconic succinic, and fumaric acids	[189]
*Bacilli strains*	Wheat rhizospheres and rock phosphate mine	Gluconic, lactic, citric, malic, succinic and propionic acids	[190]
*Pseudomonas* sp. strain AZ5, *Bacillus* sp. strain AZ17	Chickpea rhizosphere	Acetic, oxalic and gluconic acids, acetic, citric, and lactic acids	[191]
*Pseudomonas*	Glacial ice samples	Gluconic acid	[18]
*Rahnella* sp. BIHB 783	*Hippophae rhamnoides* rhizosphere	Gluconic, citric, and isocitric acids	[44]
*Acinetobacter rhizosphaerae* strain BIHB 723	Cold deserts of the trans-Himalayas	Gluconic, oxalic, 2-keto gluconic, lactic, malic, and formic acids	[192]
Fluorescent *Pseudomonas* strains	Cold deserts of the Himalayas	Gluconic acid, oxalic acid, 2-ketogluconic acid, lactic acid, succinic acid, formic acid, citric acid and malic acid	[193]
*Pseudomonas corrugata* (NRRL B-30409)	Culture Collection	Gluconic and 2-ketogluconic acids	[194]

**Table 3:** Plant growth-promoting active biomolecules released by psychrophilic/psychrotolerant phosphate solubilizing bacteria.

PPSB	Origin	Media Used	Plant Growth Enhancers	Reference
*Bacillus weihenstephanensis* MF593886	*Gentiana kurroo* Royle rhizosphere	PVK	Siderophore, HCN, ammonia, and proteases	[207]
*Pseudomonas*, *Serratia*, and *Flavobacterium*	Rhizosphere and phyllosphere of Andes Mountains and Patagonia of Chile	PVK	IAA, ACC deaminase, anti-phytopathogenic activities	[12]
*Pseudomonas*	Snow sample	NBRIP	Siderophores, cellulases, xylanases, and chitinases	[2]
*Pseudomonas koreensis* P2	Sela Lake	NBRIP	IAA, siderophore, HCN, and iron uptake	[208]
*Acinetobacter, Bacillus, Enterobacter, Klebsiella, Proteus, Pseudomonas*, and *Staphylococcus*	Renuka Lake	PVK	Ammonia, HCN, Zn solubilization, and hydrolytic enzymes	[202]
*Pseudomonas*	Antarctic soils	NBRIP	IAA, siderophores, HCN, microbial volatile organic compounds (MVOCs)	[209]
*Pseudomonas simiae* PS2	Seabuckthorn (*Hippophae rhamnoides* L.) rhizospheric soil of high altitude in trans-Himalaya	PVK	IAA, siderophore and HCN	[40]
*Pseudomonas, Bacillus, Paenibacillus, Sporosarcina, Cupriavidus* and *Paenarthrobacter*	*Lepidium meyenii* Walp. Rhizosphere	NBRIP	IAA	[210]
*Bacillus licheniformis*, *Bacillus muralis*, *Desemzia incerta*, *Paenibacillus tylopili* and *Sporosarcina globispora*	Soil and water samples	PVK	IAA, GA, siderophores, NH_3_, HCN, ACC deaminase	[211]
*Pseudomonas koreensis* and *Arthrobacter nitroguajacolicus* strainYB4	Rainfed agriculture field	PVK	IAA	[212]
*Pseudomonas*	Rhizospheric soil	NBRIP	IAA, siderophore, ACC deaminase, ammonia, NF, and antifungal compounds	[213]
*Bacillus*			HCN, ammonia, and NF	
*Stenotrophomonas*			IAA, GA, HCN, Siderophore, ACC deaminase, ammonia, NF, and antifungal compounds	
*Arthrobacter*			IAA, siderophore, ACC deaminase, ammonia, and NF	
*Acinetobacter*			IAA, GA, HCN, siderophore, ACC deaminase, ammonia, NF, and antifungal compounds	
*Exiguobacterium*			Siderophores, ammonia, and antifungal compounds	
*Providencia*			IAA, GA, HCN, siderophore, ACC deaminase, ammonia, and NF	
*Flavobacterium*			IAA, HCN, siderophore, ACC deaminase, and ammonia	
*Kocuria*			IAA, GA, HCN, and siderophore	
*Pseudomonas*	Glacial ice	NBRIP	IAA, HCN, siderophore, proteases amylases and galactosidases	[18]
*Rhizobia*	Pea nodules	PVK	IAA	[22]
*Azotobacter*, *Pseudomonas*, *Micrococcus*, and *Bacillus*	*Pennisetum clandestinum* rhizospheres	PVK	IAA and siderophores	[214]
*Pseudomonas* spp.	High altitude of the northwest Indian Himalayas	NBRIP	IAA, siderophore	[27]
*Pseudomonas* sp.	*Deschampsia antarctica* rhizosphere	PVK	IAA and EPS	[215]
*Exiguobacterium acetylicum* strain 1P	High altitude soil	NBRIP	IAA, siderophore and HCN	[216]
*Pseudomonas fragi* CS11RH1	High altitude garlic rhizosphere	NBRIP	IAA and HCN	[14]
Fluorescent *Pseudomonas*	Garhwal Himalayas region	PVK	Siderophores, antifungal activity	[15]

PPSB, PVK, and NBRIP indicate psychrophilic/psychrotolerant phosphate solubilizing bacteria medium, Pikovskaya medium, and National Botanical Research Institute Phosphate medium, respectively; IAA, HCN, GA, NF, and EPS represents indoleacetic acid, hydrogen cyanide, gibberellic acid, nitrogen fixation, and exopolysaccharides, respectively.

**Table 4:** Inoculation effect of psychrophilic/psychrotolerant phosphate biofertilizers on different crops.

Inoculated Crops	Cold Active PSB	Conditions	Agronomical Traits	References
*Arabidopsis thaliana*	*Pseudomonas* sp., *Pseudomonas proteolytica, Pseudomonas azotoformans*	Growth Chamber	Promoted overall growth such as rosette diameter, leaf area, and biomass	[20]
Tomato	Mixture of *Pseudomonas* sp. TmR5a and *Curtobacterium* sp. BmP22c (BC3)	Pot assay	Promoted the germination by 90% and significantly increased the root lengths	[12]
Tomato	*Pseudomonas*	Greenhouse	Increased germination and plantlets	[2]
Wheat	*Pseudomonas*	Paper Roll Towel Bioassay	Significantly increased root and shoot-lengths	[209]
Tomato	*Pseudomonas simiae*	Pot experiments in green shade net and open field conditions	Enhanced plant growth, increased fruit yield by 9.8% (net house) 19.8% (open field)	[40]
Chickpea, green gram, pea, and maize	*Lysinibaccilus macroides* ST-30, *P. palleroniana* N-26 and *P. jessenii* MP-1	In vitro seed germination assay	Significantly increased the germination efficiency	[253]
Red clover	*Pseudomonas, Bacillus, Paenibacillus, Sporosarcina, Cupriavidus* and *Paenarthrobacter*	Water agar plates	Increased seed germination	[210]
Rice	*Pseudomonas koreensis* and *Arthrobacter nitroguajacolicus* strainYB4	Greenhouse	Efficiently increased the biomass and P uptake	[212]
Lentil	*Pseudomonas* spp.	Temperature controlled polyhouse	Significantly increased the plant growth, grain yield, and P uptake	[27]
Barley, chickpea, pea, and maize	*Rahnella* sp.	Greenhouse	Significantly increased growth of all crops, microplot testing of the PPSB inoculum also significantly increased growth and yield of pea	[44]
Wheat	*Pseudomonas fragi* CS11RH1 (MTCC 8984)	Greenhouse	Increased the percent germination, rate of germination, biomass, and nutrient uptake	[14]
	*Pseudomonas vancouverensis*	Greenhouse	Increased germination and root and shoot length	[254]

## Data Availability

The data presented in this study are available in the main manuscript.
